# Gestational Diabetes Exposed Mesenchymal Stem Cells: Phenotypic Differences Link to Long-Term Health of Offspring

**DOI:** 10.3390/ijms262110768

**Published:** 2025-11-05

**Authors:** Mark J. Pandrich, Nishel M. Shah, Isabel Garcia Perez, Mark R. Johnson, Natasha Singh

**Affiliations:** Imperial College London, London SW7 2AZ, UK

**Keywords:** mesenchymal stem cells, mesenchymal stromal cells, gestational diabetes mellitus, diabetes in pregnancy, developmental origins of disease

## Abstract

Mesenchymal stem cells (MSCs) derived from the placenta, fetal membranes, or umbilical cord may be used to study the pathophysiology of gestational diabetes mellitus (GDM). The phenotype of MSCs may reflect fetal programming in response to the maternal milieu of a GDM pregnancy. Altered fetal programming is linked to high rates of obesity and type 2 diabetes mellitus (T2DM) in the offspring of mothers with GDM. This review discusses recent findings characterizing the phenotype of GDM-exposed MSCs (GDM-MSCs) which enhance our understanding of the mechanisms of fetal programming. It also considers how MSCs may be used as markers of long-term offspring health to test the benefit of putative interventions and highlights the need for further translational studies to clearly link the MSC phenotype to clinical parameters and interventions.

## 1. Introduction

Gestational diabetes mellitus (GDM) refers to hyperglycemia with onset during pregnancy, developing in susceptible mothers as the metabolic adaptations of pregnancy occur. Although defined as hyperglycaemia, GDM reflects a widespread metabolic derangement secondary to increased insulin resistance with or without a deficit in insulin secretion [1,2]. The altered maternal milieu seen in GDM perturbs the growth and development of the fetus, resulting in an increased risk of birth complications and a predisposition to metabolic disorders such as obesity and type 2 diabetes (T2DM) in later life [2,3,4]. The mechanisms underlying the developmental programming that mediates these adverse outcomes are incompletely understood. Examining cells of fetal origin that have been exposed to GDM may develop our understanding of these mechanisms and allow putative interventions to be tested to improve long-term health of offspring.

## 2. Mesenchymal Stem Cells

### 2.1. Definition and Fundamental Characteristics of MSCs

Mesenchymal stem cells (MSCs) are multipotent fibroblast-like cells of mesodermal origin with the potential to differentiate into adipocytes, chondrocytes, and osteoblasts [5,6,7]. MSCs may be derived from several tissues, including bone marrow (BM-MSCs), adipose tissue (AD-MSCs) and umbilical cord (UC-MSCs) [8]. The International Society for Cell & Gene Therapy (ISCT) have set out minimal criteria to define multipotent MSCs by a panel of cell-surface markers and classical trilineage mesenchymal differentiation potential [8,9]. While multipotency has been demonstrated for all MSCs in vitro, insufficient robust evidence of multipotency and self-renewal in vivo means the term mesenchymal stromal cells, also abbreviated to MSC, may be preferred [8]. MSCs may also be induced in vitro to differentiate into multiple non-mesenchymal cell types including myocytes, neural cells, and hepatocytes; however, the extent to which this happens in vivo during normal development or tissue homeostasis is contested [5,10].

The in vivo function of MSCs remain poorly understood, but they are thought to be involved in tissue repair and immune regulation [5]. Their frequent isolation from perivascular niches suggests a role in supporting angiogenesis and wound healing [5,7,11]. There is significant interest in exploiting the paracrine, immunomodulatory, and stem-cell-like properties of MSCs to develop therapeutics [5,8].

### 2.2. MSCs from Placental Membranes and Umbilical Cord

Cells of the extraembryonic mesoderm form part of the placenta, fetal membranes (amnion and chorion), and umbilical cord. Fetal MSCs can be isolated from placental tissue (P-MSCs), amnion (A-MSCs), chorion (C-MSCs), and umbilical cord (UC-MSCs), with the Wharton’s jelly of the umbilical cord a particularly rich source [12,13,14]. The ease of access and ethical acceptability of obtaining MSCs from placenta, fetal membranes, and the umbilical cord, tissues routinely discarded after birth, make them attractive sources for isolation of MSCs. Protocols initially developed to isolate MSCs for storage for future autologous therapies, noted that maternal characteristics altered the phenotype of isolated MSCs [14,15]. This phenotypic variation in MSCs may represent altered developmental programming, influenced by maternal characteristics and pregnancy conditions [12,15].

### 2.3. Placental Membrane and Umbilical Cord MSCs as a Model to Study GDM

P-, A-, C-, and UC-MSCs share a fetal MSC niche with adipose progenitors that not only give rise to fetal adipose tissue but also persist as resident progenitors within it [12]. The shared embryonic origin of MSCs with tissues implicated in the development of obesity and insulin resistance, together with their capacity in vitro to differentiate into these and other relevant lineages such as myocytes, makes MSCs a useful model to investigate how the maternal milieu of GDM may reprogram fetal tissues and increase susceptibility to metabolic disorders later in life.

## 3. Phenotype of GDM-Exposed Mesenchymal Stem Cells

### 3.1. Basic Characteristics

MSCs extracted from the placenta (P-MSCs), amnion (A-MSCs), chorion (C-MSCs) and umbilical cord (UC-MSCs), respectively, of both GDM (GDM-MSCs; GDM-P-MSCs, GDM-A-MSCs, GDM-C-MSCs, and GDM-UC-MSCs) and non-GDM, or normoglycaemic, pregnancies (NG-MSCs; NG-P-MSCs, NG-A-MSCs, GDM-C-MSCs, and GDM-UC-MSCs) demonstrate similar surface markers and the potential to differentiate into the three cell types of the mesenchymal lineage, conforming to the ISCT definition [16,17,18]. Throughout this review, the terms GDM-MSCs or NG-MSCs are used when findings have been reported across multiple pregnancy tissue sources; where results have only been demonstrated in MSCs from a particular pregnancy tissue, the source is indicated, e.g., GDM-P-MSCs.

### 3.2. Differentiation Ability

#### 3.2.1. Induction and Measurement of Differentiation

Protocols for inducing adipogenesis, osteogenesis, and chondrogenesis in MSCs in vitro are well-established and largely consistent across studies. Adipogenesis is typically induced by culture with media supplemented with isobutyl methylxanthine, indomethacin, dexamethasone, and insulin [16,18,19,20]; osteogenesis with media supplemented with ascorbic acid, dexamethasone, and β-glycerophosphate [16,19,20]; and chondrogenesis with media supplemented with insulin–transferrin–selenium (ITS), ascorbic acid, dexamethasone, sodium pyruvate, proline, and human transforming growth factor (TGF) β-1 or -3 [16,19,20]. Differentiation into adipocytes, osteoblasts, and chondrocytes is assessed by morphology and staining for characteristic products of the respective cell type: lipid droplets in adipocytes, mineral deposition in osteoblasts, and proteoglycan matrix deposition in chondrocytes. Differentiation potential refers to the capacity of a cell population to differentiate into a specific lineage, whereas differentiation efficiency describes the proportion of cells that successfully acquire the morphology or marker expression typical of that lineage in response to set induction conditions. Whilst we would expect all isolated MSCs to have trilineage mesenchymal differentiation potential, exposure to GDM in utero may alter their differentiation efficiency. Relative differentiation efficiency can be compared by measuring the intensity of staining, proportion of stain-positive cells, and by measurement of lineage specific gene and protein expression.

#### 3.2.2. Adipocyte Differentiation Potential

GDM-MSCs have higher differentiation efficiency in response to adipogenic induction, summarized in Table 1. Following induction, a higher proportion of GDM-MSCs stain positive for lipid droplets with Oil-Red-O with a higher amount of lipid per positive cell compared to NG-MSCs [16,18]. GDM-MSCs also have a greater increase in the expression of the adipocyte-associated genes peroxisome proliferator-activated receptor-γ (*PPARγ*), fatty acid-binding protein-4 (*FABP4*), and adiponectin (*ADIPOQ*) in response to adipogenic induction [16,18]. Even in the undifferentiated state, GDM-MSCs already have higher basal expression of adipocyte-associated genes, including *PPARγ*, CCAAT-enhancer-binding proteins (*CEBPβ)*, preadipocyte factor 1 (*PREF1*), and *ADIPOQ,* suggesting a predisposition toward the adipocyte lineage [16,19].

#### 3.2.3. Osteoblast Differentiation Potential

Equivalent or impaired osteogenesis of GDM-MSCs relative to NG-MSCs is reported, as summarized in Table 2 [16,17,18,21]. Mineral deposition, assessed by Von Kossa or Alizarin Red staining, following osteogenic induction is reported to be similar or reduced in GDM-MSCs relative to NG-MSCs [16,17,18,21]. No difference has been observed between GDM-MSCs and NG-MSCs in alkaline phosphatase (ALP) activity following osteogenic induction [18]. However, expression levels of osteoblast-related genes such as osteopontin (*OPN*), alkaline phosphatase (*ALP*), osteocalcin (*OC*), collagen type 1 alpha 1 (*Col1α1*), and bone sialoprotine (*BSP*) have been reported to be significantly lower in GDM-MSCs compared to NG-MSCs following osteogenic induction [17,19,21].

#### 3.2.4. Chondrocyte Differentiation Potential

Equivalent or impaired chondrogenesis of GDM-MSCs relative to NG-MSCs is reported, as summarized in Table 3 [16,17,19]. Proteoglycan rich matrix deposition, assessed by Alcian Blue staining, is reported to be similar following chondrogenic induction in GDM-UC-MSCs relative to NG-UC-MSCs [16]. Equivalent or reduced expression of chondrocyte-related genes collagen type II (*COL2A*), cartilage oligomeric matrix protein (*COMP*), fibromodulin (*FMOD*), and sex-determining region Y-box 9 (*SOX9*) has been reported in GDM-MSCs following chondrogenic induction [17,19].

#### 3.2.5. Differentiation Potential into Other Cell Types

MSCs may also be differentiated into non-mesenchymal cells. Myogenesis may be induced in MSCs in vitro by culture with media supplemented with horse serum, dexamethasone, and hydrocortisone [22]. Myogenic ability has not been directly compared between GDM-MSCs and NG-MSCs; however, one study found that metformin may program GDM-UC-MSCs towards myogenic differentiation with increased expression of proliferator-activated receptor-γ coactivator-1α (*PGC-1α*) and increased abundance of myosin heavy chain in response to myogenic induction [22].

### 3.3. Functional Comparison of GDM-MSCs to NG-MSCs

#### 3.3.1. Glucose Metabolism

The ability of GDM-MSCs to modulate glucose uptake and metabolism in response to their glucose environment is impaired, as summarized in Table 4. GDM-MSCs consume less glucose than NG-MSCs in both low- and high-glucose conditions, leaving higher concentrations in the spent media [23,24,25]. In response to insulin, GDM-MSCs show attenuated glucose uptake, reflected by a reduced rise in intracellular glucose and a smaller decline in glucose concentration in the spent media relative to NG-MSCs with the same insulin stimulus [24,25]. NG-P-MSCs increase glycogen storage in response to high-glucose conditions; however, GDM-P-MSCs show minimal glycogen storage in both low- and high-glucose conditions [24]. These differences are seen despite no difference in the expression of glucose transporters 1, 3, and 4 (*GLUT 1*, *3* and *4)* or glycogen synthase kinase-3-beta (*GSK3β)* [24].

#### 3.3.2. Mitochondrial Function

GDM-MSCs have impaired mitochondrial function compared to NG-MSCs, as summarized in Table 5. GDM-UC-MSCs have reduced gene expression of the mitochondria-related genes complex I subunit NADH-ubiquinone (*ND2*), complex V subunit (*AS8*), mitochondrial transcription factor A (*TFAM)*, peroxisome proliferator-activated receptor gamma coactivator 1-alpha (*PGC-1α),* and NADH dehydrogenase 1 beta subcomplex subunit 9 (*NDUFB9*) alongside reduced protein expression of oxidative phosphorylation system (OXPHOS) subunits ND9 and cytochrome c oxidase (COX1) and TFAM and PGC-1α [19,21]. Mitochondrial functional assays reveal impaired mitochondrial activity in GDM-UC-MSCs, with reduced intracellular ATP production relative to total reactive oxygen species (ROS) generation [21]. The basal oxygen consumption rate (OCR) of GDM-UC-MSCs is reduced compared with NG-UC-MSCs, accompanied by a reduced ability to increase respiratory capacity, as measured by OCR, in response to environmental challenge [21,25]. A slight reduction in mitochondrial staining has also been observed in GDM-P-MSCs [24].

#### 3.3.3. Oxidative Stress

Increased markers of oxidative stress have been reported in GDM-MSCs, as summarized in Table 6. GDM-MSCs compared to NG-MSCs show higher levels of ROS under normal conditions, in high-glucose conditions, and a greater increase in ROS with oxidative stress induced with hydrogen peroxide [25,26,27]. GDM-UC-MSCs have lower levels of the antioxidant glutathione (GSH) and antioxidant enzymes catalase and superoxide dismutase (SOD) [23]. There is reduced expression of genes involved in the intracellular handling of ROS, including aldehyde dehydrogenase (ALDH) family gene expression and Nrf2-mediated oxidative stress response pathway in GDM-A-MSCs [27]. GDM-MSCs have reduced aldehyde dehydrogenase function (ALDH) and a paradoxical decrease in antioxidant glutathione peroxidase (GPx) activity in response to high-glucose conditions [26,27]. GDM-UC-MSCs show higher levels of DNA damage in response to hydrogen peroxide in both low- and high-glucose conditions compared to NG-UC-MSCs [26]. Levels of malondialdehyde (MDA), a marker of lipid peroxidation by ROS, are higher in GDM-UC-MSCs compared to NG-UC-MSCs [19,23]. Premature cell death, cellular aging, and senescence are also associated with oxidative stress. GDM-MSCs have a higher rate of cell death and a reduced rate of cell proliferation compared to NG-MSCs [17,19,21,23]. GDM-MSCs have increased expression of markers of cellular senescence including β-galactosidase (β-gal), cyclin-dependent kinase inhibitors (CDKIs) p16, p21, and p27 and phosphorylated p53 expression [19,21]. Lower telomerase activity and lower expression of stem cell signature markers octamer-binding transcription factor 4 (*OCT4*), sex-determining region Y-box 2 (*SOX2*), and homeobox protein NANOG (*NANOG*) in later passages of GDM-UC-MSCs further indicate premature cellular aging and senescence [19].

#### 3.3.4. Angiogenesis

GDM-MSCs exhibit altered functions related to vascular support and angiogenesis compared to NG-MSCs, as summarized in Table 7. When co-cultured in a bilayer with human umbilical vein endothelial cells (HUVECs), GDM-UC-MSCs have higher expression of vascular endothelial growth factor (*VEGF*) and an impaired ability to maintain endothelial integrity [28]. The impact of GDM on the angiogenic properties of MSCs is inconsistent across studies. GDM-A-MSCs showed increased angiogenic capacity in tube formation assays, associated with upregulation of pro-angiogenic genes including fibroblast growth factor receptor 2 (*FGFR2),* serpin family E member 1 (*SERPINE1),* transforming growth factor beta receptor 1 (*TGFBR1)* and *VEGF* [29]. GDM-P-MSCs derived from placenta (chorionic villus) had a reduced angiogenic capacity which was associated with a downregulation in fibroblast growth factor 2 (*bFGF*) and *VEGF* [24].

#### 3.3.5. Inflammation and Immune Modulation

Current evidence suggests GDM-MSCs have a generally pro-inflammatory phenotype, as summarized in Table 8. GDM-A-MSCs have increased expression of pro-inflammatory genes, including tumor necrosis factor alpha (*TNFα*), monocyte chemoattractant protein 1 (*MCP-1*), *CD40* and cathepsin S (*CTSS*), and downregulation of anti-inflammatory interleukin-33 (*IL-33)* and prostaglandin–endoperoxide synthase 2 (*PTGS2)* alongside a reduction in prostaglandin E2 (PGE2) levels [17]. Accordingly, conditioned media from GDM-A-MSCs enhanced migration of monocytes and T lymphocytes compared to media from NG-MSCs [17]. GDM-MSCs have also been shown to have enhanced migration and invasion capacity [17,27,28]. A similar pattern of inflammatory gene expression is seen in NG-A-MSCs when stimulated with high concentrations of glucose, insulin, and palmitic acid [17].

## 4. In Vivo Relevance and Correlation of MSC Phenotype with Long-Term Health of Offspring

### 4.1. Summary of GDM-MSC Phenotype

GDM-MSCs display a predisposition toward adipogenic differentiation, alongside impaired glucose metabolism and mitochondrial function, elevated oxidative stress, altered angiogenic capacity and a shift toward a pro-inflammatory phenotype. These phenotypic differences may be of relevance to understanding the long-term consequences of GDM on the health of the offspring. If reliably linked to in vivo and long-term outcomes, the phenotype of GDM-MSCs could serve as a useful measure of outcome in studies evaluating diagnostic thresholds and treatments for GDM. To date, no studies have directly linked the phenotype of GDM-MSCs to long-term outcomes in offspring; however, inferences can be drawn from research associating the phenotypes of MSCs from pregnancies of mothers with obesity (Ob-MSCs) and normal weight (NW-MSCs) with offspring outcomes.

### 4.2. Lineage Preference

The predisposition of GDM-MSCs to differentiate along the adipocyte lineage, coupled with a reduced or unchanged capacity for chondrogenesis and osteogenesis, may have important in vivo implications for the offspring of mothers with GDM.

In vivo adipogenesis occurs in two steps, with MSCs differentiating into preadipocytes early in fetal development, with a proportion of preadipocytes then differentiating into mature adipocytes at a later point [30,31]. Different numbers of preadipocytes and mature adipocytes have been observed in lean and obese individuals, with a higher total number of adipocytes in those with obesity, which may be explained by an increased propensity of preadipocytes to become mature adipocytes in individuals who develop obesity [32,33]. The higher adipocyte differentiation efficiency observed in GDM-MSCs may demonstrate that this predisposition may be linked to GDM exposure in utero [18]. Studies of Ob-MSCs have demonstrated that the phenotype of MSC-derived adipocytes is associated with relevant short- and longer-term neonatal outcomes [12]. Greater adipogenesis of Ob-MSCs is correlated with fat mass in infants at birth [34,35,36]. The phenotype of Ob-MSC-derived adipocytes is also correlated with a greater increase in adiposity in infants at 4–6 months and 4–6 years of age [36,37]. Ob-MSC adipocyte phenotype has also been correlated with fasting glucose at 4–6 years of age [36].

As well as fat tissue, muscle tissue dysfunction is also of particular interest in understanding the development of diabetes and obesity. The sole study reporting myogenesis in GDM-MSCs only looked at the effect of metformin treatment and did not compare their response to myogenic induction or the phenotype of the resulting myocytes with NG-MSCs [22]. In Ob-MSCs, the phenotype of MSC-derived myocytes has been correlated with fat mass at birth, 4–6 months and 4–6 years of age [38,39,40].

Although less directly linked to the increased prevalence of metabolic syndrome-related conditions in offspring of mothers with GDM, the reduced osteogenic and chondrogenic differentiation potential of GDM-exposed MSCs may nonetheless have clinical relevance. For example, reduced bone strength among infants of mothers with diabetes has been reported, though this may be more closely associated with macrosomia and requires further work to link to longer-term outcomes [41,42,43].

### 4.3. Functional Differences

Exposure to hyperglycaemia during pregnancy appears to impair the ability of GDM-MSCs to regulate glucose metabolism and respond to insulin, a finding that may help establish a mechanistic link to the elevated risk of insulin resistance, prediabetes, and T2DM in offspring of mothers with GDM. The association of this altered glucose metabolism of GDM-MSCs to offspring outcomes has not been explored; however, insulin sensitivity of Ob-MSC- and NW-MSC-derived myocytes has been correlated with infant fat mass at birth and 6 months old [44,45]. Correlating glucose metabolism in GDM-MSCs to measures of infant glucose metabolism and insulin sensitivity would be of particular interest for future study.

GDM-MSCs appear to experience higher oxidative stress secondary to mitochondrial dysfunction, higher generation of ROS, and reduced expression and function of antioxidant enzymes [25,26,27]. Hyperglycaemia is known to increase oxidative stress and generation of ROS, which may propagate the development of further insulin resistance, reduce insulin production, and mediate sequalae of hyperglycaemia [26]. If the higher levels of oxidative stress seen in GDM-MSCs reflect the long-term function of infant cells, this mechanism may be an important mediator of the adverse health outcomes seen in infants of mothers with GDM. Higher levels of oxidative stress is particularly notable in GDM-MSCs when exposed to high-glucose conditions during culture, perhaps serving as a model of how GDM exposure in utero may predispose offspring to chronic disease when they later encounter an obesogenic environment [26].

MSCs are often found resident in perivascular locations [5,7,11]. MSCs resident in the placenta and fetal membranes may contribute to angiogenesis during placentation and maintenance of endothelial function by paracrine secretions and cell–cell contact mechanisms [28,46]. Altered angiogenic and vascular support functions of GDM-MSCs may underlie the increased risk of placental and vascular complications in GDM, including abnormal fetal growth, preeclampsia, and preterm birth. Supporting this, the angiogenic phenotype of GDM-MSCs correlates with offspring cord blood insulin levels—an indicator of GDM severity—and with both placental weight and neonatal adiposity [29].

GDM-MSCs appear to have a pro-inflammatory phenotype [17]. Low-grade inflammation is a typical feature of placentas exposed to GDM [47]. The pro-inflammatory phenotype of GDM-MSCs, may be a further mechanism linking GDM to placental dysfunction and its associated complications [17,47]. Furthermore, chronic inflammation is thought to be a key mediator of the development of insulin resistance in adipose and other metabolically active tissues [48,49]. The trigger of this inflammation is unknown, but a pro-inflammatory phenotype of GDM-MSCs, as adipocyte precursors, suggests a predisposition to inflammation may be programmed in utero.

### 4.4. Mechanism of GDM Programming

The mechanism of fetal programming of GDM-MSCs is suspected to be through epigenetic modifications, such as DNA methylation and histone modification [22,26]. Few studies have been conducted interrogating the epigenetic profile of GDM-MSCs. In one study, DNA methylation inhibition promoted myogenic differentiation in GDM-MSCs, supporting a role for DNA methylation in lineage preference and phenotype [22].

### 4.5. Treatments to Alter GDM-MSC Phenotype

In addition to serving as a model to investigate the mechanisms of fetal programming, MSCs provide a platform to test therapeutic interventions. Metformin, a commonly used treatment for GDM, has been investigated for its ability to mitigate GDM-induced programming of MSCs, with mixed results. In vitro activation of AMPK, a therapeutic target of metformin, increased the baseline and maximal OCR of GDM-MSCs [22]. GDM-MSCs from metformin-treated in vivo pregnancies showed no difference in adipogenesis nor improved mitochondrial function compared to diet-treated pregnancies, in fact showing significantly lower respiratory capacity, though this study may be confounded by differences in GDM severity between metformin- and diet-treated groups [50]. Another study, discussed earlier in this review, suggests that metformin may program GDM-MSCs towards myogenesis, which may be speculatively associated with improved metabolic outcomes [22].

Diet and exercise modification is also frequently recommended to treat GDM. There are no studies reporting the effect of diet or exercise modification on GDM-MSC phenotype. An exercise intervention in early pregnancy in healthy women has been shown to modify MSC phenotype and metabolism, with increased insulin sensitivity and complete glucose and fatty acid oxidation [51].

### 4.6. Comparing the Phenotype of MSCs of Different Pregnancy Tissue Sources

Although MSCs from the placenta and fetal membranes are thought to be fetal cells that derive from the same embryonic origin, the location in which they reside during pregnancy is likely to have an impact on their phenotype [14,52]. Studies comparing MSCs from different pregnancy tissue sources from healthy pregnancies have shown comparable surface markers and multilineage differentiation potential, with isolated MSCs conforming to ISCT criteria [53,54,55]. However, conforming to the minimal ISCT criteria does not guarantee similar phenotype or function. Isolated MSCs display variation in their paracrine secretions and associated gene expression by pregnancy tissue source, which may reflect different in vivo functions in their tissues of origin [52,56]. For example, MSCs isolated from amnion secrete more anti-inflammatory factors, and MSCs isolated from chorion more pro-angiogenic factors [56]. Overall analysis of gene expression and protein secretion, however, suggests that MSCs isolated from pregnancy tissues do appear to represent a distinct population, more similar to each other than to MSCs derived from adult tissues such as BM-MSCs and AD-MSCs [54,57]. Variable differentiation efficiency of MSCs by pregnancy tissue source has also been noted. C-MSCs are reported to have higher adipogenic differentiation efficiency, and A- and UC-MSCs higher osteogenic differentiation efficiency [53,55]. Furthermore, the efficacy of extraction and isolation of MSCs may differ by pregnancy tissue source [54,55,58]. Some studies have reported that apparent differences in phenotype between A-MSCs and C-MSCs may derive from difficulties in extraction, isolating, and culturing a pure sample of MSCs from the amnion, due to the close association of other cell types in this tissue [54,58]. Treatments used during different isolation protocols may also have an effect on MSC phenotype [54,58].

Although alterations in MSC phenotype associated with GDM have been reported in MSCs from all pregnancy tissue sources, few studies have directly compared these alterations in multiple tissue sources within the same study. One study that did directly compare the effect of GDM on A- and C-MSCs found that although the phenotype of each differed, GDM-MSCs from both tissues exhibited increased expression of adipocyte-associated genes compared to their NG-MSC counterparts [16]. Similarly, studies investigating MSC phenotype in association with maternal obesity have reported consistent results between MSCs from different pregnancy tissues [59]. More direct comparisons of MSCs from multiple pregnancy tissue sources would help to determine which GDM-induced alterations are generalizable across tissues and which are tissue-specific, enabling better comparison and integration of findings from studies conducted to date. Additionally, GDM-induced alterations to GDM phenotypes that are generalizable across tissues sources would perhaps be more likely to have a persisting impact on offspring health. It is also notable that all studies to date that have linked MSC phenotype with long-term offspring outcomes have done so using UC-MSCs [12].

### 4.7. Limitations of Current Studies

A significant challenge in investigating the effect of GDM on MSC phenotype is the heterogeneity in the diagnosis and treatment of GDM. GDM is not a binary variable and diagnostic criteria and treatment approaches vary widely across settings [2]. Most studies of MSC phenotypes neither report the criteria used to diagnose GDM, nor consider these results in subsequent analyses. The severity of hyperglycemia, or other associated metabolic disturbance, is likely an important determinant of any GDM-induced fetal programming. Therefore, the results of GDM screening, indicating the severity of hyperglycaemia at the time of diagnosis, and the subsequent treatment and glycemic control during pregnancy, should both be reported in future studies and considered as significant variables in the resulting phenotype of MSCs. Similarly, sparse data are often provided about maternal characteristics. Maternal factors, notably obesity, can have a significant impact on MSC phenotype [36]. Reporting, and where possible controlling or matching for maternal BMI would help account for the competing effects of these exposures on MSC phenotype.

## 5. Future Directions

Research into the effects of GDM on MSC phenotype remains limited, with only a small number of studies conducted typically involving small cohorts. Prospective cohort studies and ideally randomized studies are needed to establish whether the associated phenotype can be modified by intervention during pregnancy. Ideally, future studies should examine the phenotype of GDM-MSCs derived from multiple pregnancy tissues sources and link to long-term follow-up of offspring. Comparison of pregnancy tissue-derived MSCs to cells from offspring later in life, such as adipose-derived MSCs (AD-MSCs), could provide further insights into whether GDM-induced alterations persist postnatally. Further characterization of the phenotype of GDM-MSCs is also needed, including studies of functional differences following adipo- and myogenesis, and of the epigenetic mechanisms that underpin the altered phenotype.

## Figures and Tables

**Table 1:** Summary of phenotypes of gestational diabetes mellitus exposed mesenchymal stem cells (GDM-MSCs) following adipogenic induction in comparison to mesenchymal stem cells from normoglycaemic pregnancies (NG-MSCs). ↑ indicates increase, **°** indicates not reported.

Measure of Differentiation	GDM-MSCs Pregnancy Tissue Source			
	GDM-P-MSCs	GDM-A-MSCs	GDM-C-MSCs	GDM-UC-MSCs
Proportion cells lipid droplet positive	↑	↑	°	↑
Lipid content per positive cell	°	°	°	↑
Expression of characteristic genes	↑	↑	°	↑

**Table 2:** Summary of phenotypes of GDM-MSCs following osteogenic induction in comparison to NG-MSCs. ↓ indicates decrease, ↔ indicates no difference, ° indicates not reported.

Measure of Differentiation	GDM-MSCs Pregnancy Tissue Source			
	GDM-P-MSCs	GDM-A-MSCs	GDM-C-MSCs	GDM-UC-MSCs
Staining for mineral deposition	°	↓/↔	↔	↔
Alkaline Phosphatase activity	°	°	°	↔
Expression of characteristic genes	°	↓	°	↓

**Table 3:** Summary of phenotypes of GDM-MSCs following chondrogenic induction in comparison to NG-MSCs. ↓ indicates decrease, ↔ indicates no difference, ° indicates not reported.

Measure of Differentiation	GDM-MSCs Pregnancy Tissue Source			
	GDM-P-MSCs	GDM-A-MSCs	GDM-C-MSCs	GDM-UC-MSCs
Staining for proteoglycan matrix	°	↔	↔	°
Expression of characteristic genes	°	↔	°	↓

**Table 4:** Summary of differences in glucose metabolism of GDM-MSC. ↓ indicates decrease, ↔ indicates no difference, ° indicates not reported.

Glucose Metabolism	GDM-MSCs Pregnancy Tissue Source			
	GDM-P-MSCs	GDM-A-MSCs	GDM-C-MSCs	GDM-UC-MSCs
Glucose uptake/consumption	↓	°	°	↓
Insulin sensitivity	↓	°	°	↓
Glycogen production	↓	°	°	°
Expression of related genes	↔	°	°	°

**Table 5:** Summary of differences in mitochondrial function of GDM-MSCs. ↓ indicates decrease, ° indicates not reported.

Mitochondrial Function	GDM-MSCs Pregnancy Tissue Source			
	GDM-P-MSCs	GDM-A-MSCs	GDM-C-MSCs	GDM-UC-MSCs
Expression of related genes	°	°	°	↓
Expression of related proteins	°	°	°	↓
Respiratory capacity	°	°	°	↓
Mitochondrial staining	↓	°	°	°

**Table 6:** Summary of differences in oxidative stress of GDM-MSCs. ↑ indicates increase, ↓ indicates decrease, ° indicates not reported.

Measure of Oxidative Stress	GDM-MSCs Pregnancy Tissue Source			
	GDM-P-MSCs	GDM-A-MSCs	GDM-C-MSCs	GDM-UC-MSCs
Generation of ROS	°	↑	°	↑
Expression of antioxidants proteins	°	°	°	↓
Antioxidant enzyme function	°	↓	°	↓
Expression of related genes	°	↓	°	°
DNA damage	°	°	°	↑
Malondialdehyde levels (MDA)	°	°	°	↑
Markers of cell death	°	↑	°	↑
Markers of cellular senescence	°	°	°	↑

**Table 7:** Summary of differences in angiogenesis of GDM-MSC. ↑ indicates increase, ↓ indicates decrease, ° indicates not reported.

Measure of Angiogenesis	GDM-MSCs Pregnancy Tissue Source			
	GDM-P-MSCs	GDM-A-MSCs	GDM-C-MSCs	GDM-UC-MSCs
Angiogenesis (assay)	↓	↑	°	°
Support for endothelial integrity	°	°	°	↓
Expression of related genes	↓	↑	°	°

**Table 8:** Summary of inflammatory phenotype of GDM-MSC. ↑ indicates increase, ↓ indicates decrease, ° indicates not reported.

Measure of Inflammation	GDM-MSCs Pregnancy Tissue Source			
	GDM-P-MSCs	GDM-A-MSCs	GDM-C-MSCs	GDM-UC-MSCs
Expression of pro-inflammatory genes	°	↑	°	°
Expression of anti-inflammatory genes	°	↓	°	°
Enhanced migration and invasion	°	↑	°	↑

## Data Availability

No new data were created or analyzed in this study. Data sharing is not applicable to this article.
